# Diagnostic Performance of Greyscale Ultrasound in Detecting Fatty Liver Disease in a Type 2 Diabetes Population Using FibroScan as the Reference Standard

**DOI:** 10.7759/cureus.40756

**Published:** 2023-06-21

**Authors:** Yaw A Wiafe, Ijeoma C Anyitey-Kokor, Richmond A Nmai, Mary Afihene, Lewis R Roberts

**Affiliations:** 1 Department of Medical Diagnostics, Kwame Nkrumah University of Science and Technology, Kumasi, GHA; 2 School of Medicine and Dentistry, Kwame Nkrumah University of Science and Technology, Kumasi, GHA; 3 Division of Gastroenterology and Hepatology, Mayo Clinic College of Medicine, Rochester, USA

**Keywords:** fatty liver disease, fibroscan, transient elastography, non-alcoholic fatty liver disease, nafld, hepatic steatosis, types 2 diabetes, b-mode ultrasound, transient elastography (fibroscan)

## Abstract

Introduction

Brightness mode ultrasound (B-mode US) and FibroScan (Echosens, Paris, France) are the two ultrasound methods often recommended for screening non-alcoholic fatty liver disease (NAFLD) in persons with type 2 diabetes mellitus (T2DM). This study assessed the diagnostic performance of B-mode US using FibroScan as the reference standard.

Methods

Persons with a known history of T2DM were invited to screen for NAFLD using B-mode US and FibroScan on separate days within a one-month period. Assessors of B-mode US and FibroScan were blinded to each other’s findings. Both B-mode US and FibroScan independently assessed and graded each participant for the presence of NAFLD. Using the diagnostic test findings of FibroScan as a reference standard, the sensitivity and specificity of B-mode US were analyzed. The area under the receiver operating characteristic curve (AUROC) was analyzed using Jamovi (version 2.3.21). A multinomial logistic regression of the B-mode US and FibroScan in predicting NAFLD grade was also analyzed.

Results

A total of 171 participants were assessed. B-mode US detected NAFLD in T2DM patients with 63.6% sensitivity, 65.6% specificity, and 0.646 AUROC. Sensitivity and specificity in overweight and obese participants were 36-43% and 76-85%, respectively. Multinomial logistic regression demonstrated an insignificant statistical relationship between FibroScan and B-mode US in predicting grade 1 steatosis (p-value = 0.397), which was significantly affected by a higher BMI (p-value = 0.034) rather than a higher liver fibrosis level (p-value = 0.941). The logistic regression further showed a significant relationship between B-mode US and FibroScan in predicting steatosis grade 2 (p-value = 0.045) and grade 3 (p-value < 0.001), which was not significantly affected by BMI (p-value = 0.091).

Conclusion

B-mode US can replace FibroScan for severe steatosis; however, it cannot be used to screen for NAFLD in T2DM patients due to lower sensitivity for early detection in the overweight.

## Introduction

Non-alcoholic fatty liver disease (NAFLD) is on the rise in both developed and developing countries because of inadequate physical activity and a shift toward high-fat and high-sugar diets [1]. It often co-exists with other metabolic diseases like obesity and type 2 diabetes mellitus (T2DM) [2], which escalates the progression of NAFLD to steatohepatitis (NASH) and cirrhosis [1,3]. Early detection of NAFLD may enable patients to make lifestyle modifications to minimize adverse outcomes [4].

Although people with T2DM have a higher prevalence of NAFLD than the general population, the diagnostic performance of brightness mode ultrasound (B-mode US) in this population has not been determined using transient elastography as the reference standard. Recent papers have recommended B-mode US and transient elastography for screening people with T2DM because of the high prevalence of NAFLD amongst this population [5-7].

The B-mode US technique is the initial imaging modality of choice for assessing NAFLD. B-mode US uses a relatively subjective interpretation in grading NAFLD [8]. This is mainly based on greyscale liver brightness, the contrast between the liver and kidney, and visualization of the gallbladder wall, intrahepatic vessels, and the diaphragm [9]. Although B-mode US is less sensitive for detecting below 5% steatosis, it is at par with computed tomography (CT) and magnetic resonance imaging (MRI) in detecting moderate-to-severe steatosis [9]. This makes B-mode US a preferred option because of its affordability, accessibility, and lack of ionizing radiation.

In recent times, alternative ultrasound methods for quantitative grading of NAFLD have emerged with improved sensitivity for detecting low-grade steatosis [10]. One of them is the transient elastography technique, which uses amplitude mode ultrasound for assessing liver stiffness. Ultrasound elastography analyzes the mechanical properties of liver tissue by monitoring the feedback from transmitted acoustic energy. Because of the direct relationship between tissue stiffness and shear wave propagation velocity [11,12], the technology uses an increase in velocity to estimate liver fibrosis. The technology also quantifies hepatic steatosis by measuring the energy loss as the sound wave passes through the medium, which is known as the controlled attenuation parameter (CAP). This dual technology is available in the FibroScan system manufactured by Echosens (Paris, France). Several studies using liver biopsy as the reference standard have reported that the CAP performs well in grading liver steatosis [13,14].

However, the relationship between B-mode US and FibroScan in assessing liver steatosis is poorly understood. Kamali et al. [15] assessed the sensitivity and specificity of B-mode US using FibroScan as the reference standard, but in a smaller sample population. Their study also did not account for body mass index (BMI) and metabolic diseases that are known to affect the performance of both B-mode US and FibroScan [15]. This study was therefore conducted to investigate the diagnostic performance of B-mode US in comparison to FibroScan in a T2DM population. It also assessed the impact of BMI and liver fibrosis on the relationship between B-mode US and FibroScan.

## Materials and methods

Assessor blinded cross-sectional study design was used in conducting this study. The study was conducted at Kwadaso Seventh-Day Adventist Hospital in Kumasi, Ghana. Patients with a known history of having T2DM (based on fasting glucose or glycosylated hemoglobin history) were invited for voluntary participation in this study. Patients below the consenting age of 18 years were excluded from the study. A total of 171 consenting individuals with T2DM participated in the study.

Ethical consideration

Ethical approval for the study was granted by the Committee on Human Research and Publication Ethics (CHRPE) at the Kwame Nkrumah University of Science and Technology, Kumasi, Ghana (approval no.: CHRPE/AP/772/22). Participants were provided with a detailed explanation of the study protocol and assured of their confidentiality and the right to decline participation. All participants provided written informed consent.

Data collection and transient elastography measurements

The weight and height of the participants were measured in kilograms (kg) and centimeters (cm), respectively, and body mass indices were calculated in kilograms per meter square (kg/m2).

B-mode US and FibroScans were performed by two experienced sonographers with over 10 years of clinical ultrasound experience who were blinded to each other’s findings as well as the study participants’ clinical and anthropometric data. All abdominal ultrasound scans were performed after an appropriate fasting period of about six hours. Using a Mindray DC30 (Mindray Bio-Medical Electronics Co. Ltd., Shenzhen, China) ultrasound machine with a 3-5 MHz transducer, B-mode US images of the liver were obtained, including sagittal images in the midclavicular plane demonstrating the liver and the kidney, sagittal images demonstrating the liver parenchyma and the diaphragm, sagittal images of the gallbladder, and transverse images demonstrating the portal vein and the confluence of the intrahepatic veins at the inferior vena cava (IVC). The B-mode US grading of steatosis was based on liver echogenicity in comparison with the right kidney and the visualization of the gallbladder wall, intrahepatic vessels, and diaphragm. Grade 0 referred to normal liver echogenicity in comparison to the right kidney. Grade 1 referred to a mild increase in liver parenchymal echogenicity with adequate visualization of the gallbladder walls, intrahepatic vessels, and diaphragm. Grade 2 referred to a remarkable increase in liver parenchymal echogenicity in comparison to the right kidney with reduced visualization of the gallbladder wall, intrahepatic vessels, and diaphragm. Grade 3 referred to a marked increase in liver parenchymal echogenicity in comparison to the right kidney with non-visualization of intrahepatic vessels in the far field as well as the diaphragm.

A FibroScan system manufactured by Echosens, France, equipped with M and XL probes was used in assessing liver steatosis. The machine calculated the CAP score of each patient in decibels per m (dB/m) from the right hypochondriac region of the abdomen. The CAP score of each participant was used to determine the amount of hepatic steatosis present. CAP cutoff values indicated liver steatosis and ranged from S0 (no steatosis) to S3 (severe steatosis). S0 referred to a CAP score of less than 238 dB/m, S1 CAP values ranged from 238.0 to 259.0 dB/m, S2 CAP values ranged from 259.0 to 291.0 dB/m, and S3 CAP values ranged from 291.0 to 400.0 dB/m [16].

Data analysis

Microsoft Excel 2016 (Microsoft Corporation, Redmond, WA) was used for data entry and analysis was performed using Jamovi (version 2.3.21). Categorical data were presented as frequency (percentages). The normality of continuous variables was tested by the Kolmogorov-Smirnov test.

Diagnostic test findings of FibroScan on whether NAFLD was present or absent were used to determine B-mode US test findings as either true positive, true negative, false positive, or false negative. Jamovi (version 2.3.2.1) statistical analysis software was used in analyzing the area under the receiver operating characteristic curve (AUROC). Multiple regression analysis was done to determine the causality of FibroScan and B-mode US agreement on steatosis grades. A p-value of <0.05 was regarded as the significance level.

## Results

Demographics

Table 1 shows the demographic characteristics of the study participants. There were a total of 171 participants, but one participant was eliminated due to incomplete data. There was a significantly larger female population of 78% (n = 134). The mean age of participants was 59.5 years, with 25th and 75th percentiles of 52.0 and 60.0 years, respectively. There was a fair distribution of the study participants amongst the normal weight, overweight, and obese categories (Table 1). The CAP score of 53.2% of participants was above 237 dB/m, which is indicative of NAFLD [15], with 46.8% having a CAP score of less than or equal to 237 dB/m.

**Table 1:** Demographic characteristics of study participants BMI: body mass index; IQR: interquartile range; TE: transient elastography.

Variable	Categories	Frequency	Percentage
Gender	Male	37	21.8%
	Female	133	78.2%
Age	<30	9	5.3%
	30-39	22	12.9%
	40-49	54	31.8%
	50-59	55	32.4%
	60-69	22	12.9%
	70-79	8	4.7%
BMI	Normal weight (18.5-24.9)	61	36.0%
	Overweight (25-29.9)	55	32.3%
	Obese (≥30)	54	31.7%
Fibrosis score (TE)	F0	156	91.8%
	F1	13	7.6%
	F2	1	0.6%
FibroScan IQR	<10%	116	68.2%
	10-19%	44	25.9%
	20-29%	6	3.5%
	30-39%	2	1.2%
	40-49%	2	1.2%

Diagnostic test data for FibroScan versus B-mode US results on NAFLD

Table 2 shows the positive and negative diagnostic test results of FibroScan versus B‑mode US. The vertical readings represent FibroScan as the gold standard, while the horizontal readings represent B-mode US.

**Table 2:** Diagnostic test results of FibroScan and B-mode US B-mode US: brightness mode ultrasound.

	Gold positive	Gold negative	Total
Test positive	39	21	60
Test negative	40	70	110
Total	79	91	170

Receiver operating characteristic curve

**Figure 1:**
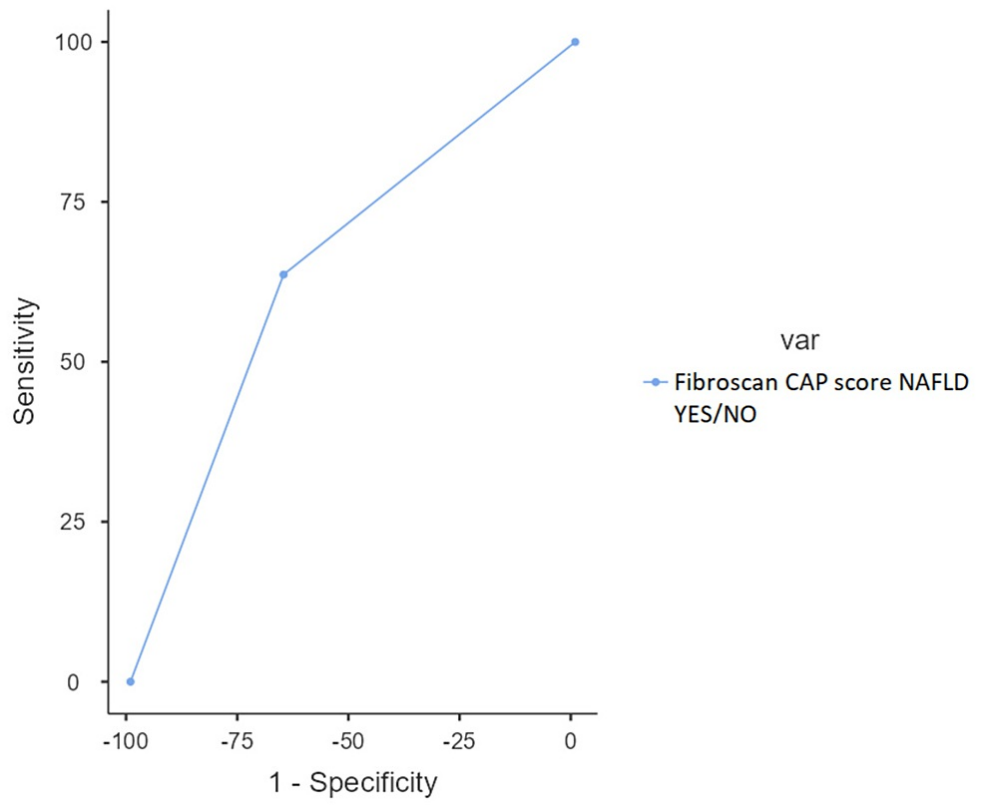
Receiver operating characteristics (ROC) curve on a YES or NO agreement on NAFLD CAP: controlled attenuation parameter; NAFLD; non-alcoholic fatty liver disease.

Figure 1 shows the receiver operating characteristic (ROC) curve on the accuracy of B-mode US in predicting NAFLD using FibroScan as the gold standard.

The area under the curve (AUC) for determination of the diagnostic accuracy of B-mode US was 0.646 with a sensitivity of 63.64% and specificity of 65.57% (Table 3). The positive and negative predictive values were 77% and 50% (see Table 3), and therefore not of significant diagnostic accuracy for the determination of NAFLD.

**Table 3:** B-mode US accuracy on NAFLD B-mode US: brightness mode ultrasound; NAFLD; non-alcoholic fatty liver disease; PPV: positive predictive value; NPV: negative predictive value; AUC: area under the curve.

Cutpoint	Sensitivity (%)	Specificity (%)	PPV (%)	NPV (%)	Youden's index	AUC	Metric score
2	63.64%	65.57%	76.92%	50%	0.292	0.646	1.29

Diagnostic accuracy of B-mode US in normal weight, overweight, and obese patients

Table 4 shows the diagnostic performance of B-mode US stratified by BMI ranges. This includes the number of NAFLD and non-NAFLD cases determined by FibroScan, the number of participants who were diagnosed by FibroScan CAP score as having NAFLD, and the results of B-mode US. The sensitivity, specificity, accuracy, and negative and positive predictive values of B-mode US in the various BMI ranges are shown in Table 4.

**Table 4:** Diagnostic performance analysis stratified by body mass index B-mode US: brightness mode ultrasound; NAFLD; non-alcoholic fatty liver disease.

Descriptive data on diagnostic accuracy	Normal weight	Overweight	Obese
Population	61	55	54
Diseased (NAFLD)	40	25	14
Healthy (no NAFLD)	21	30	40
Positive tests	33	16	12
Negative tests	28	39	42
True test	38	32	40
Wrong test	23	23	14
Diagnostic performance of B-mode US	Normal weight	Overweight	Obese
Sensitivity	62.5%	36.0%	42.9%
Specificity	61.9%	76.7%	85.0%
Accuracy	62.3%	58.2%	74.1%
Prevalence	65.6%	45.5%	25.9%
Positive predictive value	75.8%	56.3%	50.0%
Negative predictive value	46.4%	59.0%	81.0%

Multinomial logistic regression of factors affecting agreement on NAFLD grading

Table 5 shows the multinomial logistic regression analysis of the potential effect of BMI and liver fibrosis levels in the grading of NAFLD. It shows that there was an insignificant statistical relationship between FibroScan and B-mode US in predicting grade 1 steatosis (p-value = 0.397). It further shows increasing BMI (overweight and obesity) as the significant factor affecting the relationship between the two methods with p-values of 0.046 for overweight and 0.034 for obese individuals. The logistic regression further shows a slight statistically significant relationship between B-mode US and FibroScan in predicting grade 2 steatosis at a p-value of 0.045, which was not affected by overweight but significantly affected by obesity. A stronger statistical significance was found between the relationship of B-mode US and FibroScan in predicting grade 3 steatosis (p-value < 0.001), which is not affected by BMI.

**Table 5:** Multivariate logistic regression on the prediction of NAFLD grade S0: steatosis grade 0; S1: steatosis grade 1; S2: steatosis grade 2; S3: steatosis grade 3; F0: fibrosis grade 0; F1: fibrosis grade 1; F2: fibrosis grade 2; KPa: kilopascal; SE: standard error; Z: Z score; p: P-value; NAFLD; non-alcoholic fatty liver disease.

FibroScan (fatty liver grading)	Predictor	Estimate	SE	Z	p
S1-S0	Intercept	-2.012	0.463	-4.3425	
	B-mode ultrasound grading	0.280	0.358	0.7812	0.435
	Body mass index:				
	Overweight – normal weight	1.101	0.553	1.9913	0.046
	Obese – normal weight	1.297	0.612	2.1181	0.034
	Fibrosis grade (KPa):				
	F1 – F0	1.329	0.881	1.5086	0.131
	F2 – F 0	-2.725	36.586	-0.0745	0.941
S2-S0	Intercept	-2.271	0.487	-4.6637	
	B-mode ultrasound grading	0.714	0.356	2.0059	0.045
	Body mass index:				
	Overweight – normal weight	0.734	0.578	1.2700	0.204
	Obese – normal weight	1.244	0.613	2.0284	0.043
	Fibrosis grade (KPa):				
	F1 – F0	1.317	0.893	1.4748	0.140
	F2 – F0	-4.025	16.931	-0.2377	0.812
S3-S0	Intercept	-3.997	0.697	-5.7321	
	B-mode ultrasound grading	2.235	0.414	5.4001	
	Body mass index:				
	Overweight – normal weight	0.220	0.714	0.3082	0.758
	Obese – normal weight	1.173	0.695	1.6876	0.091
	Fibrosis grade (KPa):				
	F1 – F0	1.463	0.945	1.5480	0.122
	F2 – F0	11.461	672.419	0.0170	0.986

## Discussion

This study investigated the diagnostic performance of B-mode US in a T2DM population using the FibroScan CAP score as the reference standard. B-mode US is the first-line modality for screening recommended by several guidelines [5,6]. Biopsy remains the gold standard for definitive diagnosis; however, biopsies come with procedural challenges, such as incorrect or inadequate sampling and variations in histopathological interpretations [5,17]. Transient elastography or FibroScan is currently a recognized screening method for the early diagnosis of NAFLD [5-7], making it an acceptable alternative to biopsy.

The diagnostic accuracy of FibroScan for detecting NAFLD has been reported as having 68.8% sensitivity and 82.2% specificity in comparison with histology [18]. However, B-mode US is still regarded as the first-line imaging screening tool for NAFLD before transient elastography due to its affordability and accessibility [7]. The sensitivity and specificity of B-mode US have been reported to range from 53% to 76% and 76% to 96%, respectively [19,20].

Only one study has shown the sensitivity and specificity of B-mode using transient elastography as the gold standard [15]. In this first study, the AUROC for B-mode US was 0.709 with a sensitivity and specificity of 72.9% and 68.9%, respectively. However, there was no evaluation of the effects of BMI and diabetes, which earlier studies reported as important factors in CAP scores [18,21,22]. Chan et al. [21] compared tissue histology to CAP scores of obese and non-obese patients and found that the optimal AUROC CAP cutoffs for estimating steatosis grades were higher in the non-obese than in the obese. Jung et al. [22] also found that the CAP scores of their study population were significantly affected by the steatosis grade and the BMI. Karlas et al. [18] reported that a patient's diabetes can have a significant effect on CAP results, indicating the need to consider BMI and diabetes status in the workup of clients for NAFLD.

In this study, we obtained an AUROC of 0.646, with a specificity and sensitivity of 63.6% and 65.6%, respectively. This diagnostic performance is within the range of reported accuracy from earlier studies that compared B-mode US to either histology or alternative imaging methods such as proton magnetic resonance spectrometry [19,20]. This present study obtained a lower sensitivity and specificity than the first study conducted by Kamali et al. [15], probably because of differences in BMI and T2DM, which were not stated in their study. The findings on the sensitivity of B-mode US in this study imply that it has the ability to correctly identify 63.6% of T2DM persons who are truly NAFLD positive, suggesting that it could also miss nearly 37% of true positive NAFLD cases. The specificity results also imply that B-mode US can correctly determine 65.6% of T2DM cases who are truly NAFLD negative, with the chance of misdiagnosing about 35% of NAFLD negative cases. It is also worth noting that the NAFLD positive test rate by B-mode US was 64.7% in this study, which is 20% higher than the actual disease rate in the population (Table 3), as determined by transient elastography. This is a specificity problem of B-mode US, which resulted in 20% of healthy livers being diagnosed as NAFLD positive. In terms of sensitivity, B-mode US could also not identify about 10% of true positive NAFLD cases.

Further stratification of study participants showed the impact of BMI in detecting NAFLD in this population (Table 5). There was a decline in sensitivity by approximately 20-25% in the overweight and the obese. On the other hand, there was a consistent rise in specificity by 15-25% in the overweight and obese (Table 5). This implies that overweight and obesity are largely accountable for the 20% overestimation, and 10% underestimation of NAFLD in persons with T2DM.

In a multinomial logistic regression analysis, we found that there was a poor relationship between B-mode US and FibroScan in predicting grade 1 steatosis, which was significantly due to higher BMI but not the level of fibrosis (Table 5). Conversely, there was a statistically significant relationship between B-mode US and FibroScan in predicting steatosis grade 2 (p = 0.045) and grade 3 (p < 0.001). The effect of higher BMI was very minimal on the prediction of grade 2 steatosis and totally insignificant on the prediction of grade 3 steatosis.

The main limitation of this study is that B-mode US was assessed with reference to an imperfect gold standard, which can also be affected by sound transmission pitfalls associated with the body habitus of a patient. However, in this study over 98% of FibroScan interquartile ranges were below 40%, which indicates high validity [23].

## Conclusions

Using FibroScan as the reference standard, B-mode US showed a sensitivity of 63.6% and a specificity of 65.6%, with AUROC of 0.646 in detecting NAFLD amongst persons with T2DM. There was lower sensitivity of 36-43% with higher specificity of 76-85% in overweight and obese persons. However, although higher BMI affects the sensitivity of B-mode US in detecting NAFLD, it does not significantly affect the prediction of severe steatosis. This implies that while B-mode US could be used as an alternative to FibroScan in detecting grade 3 steatosis, it cannot be used for detecting early steatosis, especially in overweight and obese persons. It is therefore not suitable for screening NAFLD in persons with T2DM.
